# Cholesterol Levels in Genetically Determined Familial Hypercholesterolaemia in Russian Karelia

**DOI:** 10.1155/2017/9375818

**Published:** 2017-03-28

**Authors:** V. A. Korneva, T. Yu. Kuznetsova, T. Yu. Bogoslovskaya, D. S. Polyakov, V. B. Vasilyev, A. V. Orlov, M. Yu. Mandelshtam

**Affiliations:** ^1^Department of Faculty Therapy, Phthisiology, Infectious Diseases and Epidemiology, Institute of Medicine, Petrozavodsk State University, 33 Lenin Str., Petrozavodsk, Republic of Karelia 185910, Russia; ^2^Department of Molecular Genetics, Institute of Experimental Medicine, 12 Pavlov Street, St. Petersburg 197376, Russia; ^3^St. Petersburg State University, Universitetskaya Nab. 7/9, St. Petersburg 199034, Russia; ^4^Department of Analysis of Competitive Systems, National Research Nuclear University “MEPhI”, Kashirskoye Shosse 31, Moscow 115409, Russia; ^5^Department of Biochemistry, St. Petersburg State University, Universitetskaya Nab. 7/9, St. Petersburg 199034, Russia

## Abstract

Familial hypercholesterolaemia (FH) is a rare disease that tends to be diagnosed lately. In Russia, the genetic and phenotypic characteristics of the disease are not well defined. We investigated 102 patients with definite FH. In 52 of these patients (50.9%) genetic analysis was performed, revealing pathogenic mutations of the low density lipoprotein (LDL) receptor gene in 22 patients. We report here five mutations of the LDL receptor gene found in the Karelian FH sample for the first time. The detection rate of mutations in definite FH patients was 42.3%. Two groups of patients with a definite diagnosis of FH according to the Dutch Lipid Clinic Network criteria were compared: the first group had putatively functionally important LDL receptor gene mutations, while in the second group LDL receptor gene mutations were excluded by single-strand conformation polymorphism analysis. Total and LDL cholesterol levels were higher in the group with LDL receptor mutations compared to the mutation-free population. The frequency of mutations in patients with LDL cholesterol > 6.5 mmol/L was more than 3 times higher than that in patients with LDL < 6.5 mmol/L. Total and LDL cholesterol levels and the frequency of coronary heart disease and myocardial infarction were higher in the group with definite FH compared to groups with probable and possible FH. Cholesterol figures in FH patients of different age and sex from the Karelian population were comparable.

## 1. Introduction

Familial hypercholesterolaemia (FH) is an autosomal codominant disorder characterized by raised concentrations of low density lipoprotein (LDL) cholesterol in blood and an average 3–13 times greater risk of premature atherosclerotic cardiovascular disease, compared with individuals with normal blood concentrations of LDL cholesterol [1–3]. This disease leads to accelerated development of atherosclerotic lesions in the blood vessels, especially the coronary arteries, and clinical manifestations of ischemic heart disease (IHD) in young people and even children [4]. FH was first described in 1938, but the number of publications devoted to the FH is constantly increasing, reflecting the interest in this problem [5]. In Russia, the interrelation between LDL cholesterol levels and the detection of mutations had never been studied.

The aim of this study was to determine the features of FH in Karelia, Russia (genetic peculiarities; changes in lipid parameters in patients with definite FH) and to identify the LDL levels associated with mutation of the LDL receptor in patients with FH in Karelia.

## 2. Methods

We created a registry of patients with FH in our Republic during 2004–2016, and this now includes 254 patients. For its creation we selected patients with total cholesterol (TC) levels > 7.9 mmol/L and LDL cholesterol levels > 4.9 mmol/L. Patients with triglyceride levels > 1.9 mmol/L were not included in the study. After exclusion of secondary dyslipidemia, we diagnosed FH according to the Dutch Lipid Clinic Network (DLCN) criteria.

Clinical and biochemical features of patients with FH are presented in Table 1. We established the diagnosis of FH as “definite” if the total DLCN score was >8 (102 patients), “probable” if the score was in the range 6–8 (26 patients), and “possible” if the score was >3 (126 patients). FH was considered to be excluded if the total score was less than 3. The diagnosis took account of the presence of a family history burdened by cardiovascular disease (early development of cardiovascular disease in first-degree relatives in men before the age of 55 years and women before 60 years), the presence of coronary artery disease or atherosclerotic lesions, and the presence of first-degree relatives with hypercholesterolaemia and looked for the characteristic stigmata of FH (tendon xanthomas; lipid arc of the cornea, in individuals below the age of 45 years).

In this analysis only data from patients with definite FH are presented.

Lipid profile was estimated using the enzymatic colorimetric method. Clinical examination included the determination of glucose, creatinine, urea, thyroid hormones, total bilirubin, electrocardiography, Holter electrocardiography, echocardiography, and a scan of the brachiocephalic arteries and arteries of the lower extremities. Stress tests and coronary angiography were performed only if the patients had the relevant indication.

For DNA extraction from leukocytes of peripheral blood we used the method of Kunkel et al. [6] with the Bell et al. modification [7] for small amounts of blood. For DNA purification, we used 700–1000 *μ*L of frozen blood. Polymerase chain reaction (PCR) analysis was carried out using the “Terzic” PCR device (“DNA Technology,” Moscow) in 0.5 mL microtubes. For amplification of all exons of the gene we used primers synthesized from published sequences [8, 9]. The principal method for mutation detection was automated detection of single-strand conformation polymorphism analysis followed by direct PCR-amplified DNA sequencing. We performed genetic analysis in 114 patients from our registry, 52 of whom were diagnosed with definite FH.

### 2.1. Determination of the Reference Intervals

Since the probability distribution of the indicators in our sample differed significantly from the statistical normal distribution, the lower and upper limits of the reference interval for each metric were determined as 2.5th and 97.5th percentiles, respectively. Using this approach, the reference interval included 95% of all baseline samples, as when using a 95% confidence interval indicator, applied to variables whose distribution shows no statistically significant differences with the normal law. All the calculations were performed using the program Statistica V. 10.

## 3. Results

Genetic analysis was performed in 52 patients with definite FH from this group, revealing a mutation in the LDL receptor gene in 22 of them. Thus, the detection rate of mutations in definite FH patients was 42.3%.

The following features of the genetic “profile” of FH in Karelia were established. The* APOB* R3500Q mutation common for FH in Europe was not found in FH patients from Karelia in the studied sample [10]. Considering the epidemiology of FH in the Petrozavodsk population, we can conclude that the FH cohort is very heterogeneous, showing no predominant FH-causing mutations. We identified 14 mutations in the LDL receptor gene that are probably causative for FH development, namely, c.192del10/ins8, c.195–196insT,* p. (Ser206Arg)*, c.925–931del7,* p. (Ser447Cys)*,* p. (Leu511Ser)*, c.1686del8/insT,* p. (Leu646Ile), *c.2191delG, c.313+2T>G,* p. (Cys82Ser), p. (Glu408Lys), p. (Trp443Arg)*, and* p. (Trp620Ser)*. Of these mutations, 4 have been previously described in other populations in the world—c.925–931del7,* p. (Leu511Ser)*,* p. (Glu408Lys),* and* p. (Trp443Arg)*—whereas the remaining 10 were found only in the Karelian FH sample. Nine mutations were considered in a previous paper of ours [10] and five* p. (Cys82Ser), p. (Glu408Lys), p. (Trp443Arg)*,* p. (Trp620Ser)*, and c.313+2T>G are reported here for the Karelian population for the first time (Table 2).

All mutations presumed to be important for the development of disease were found in the unique families and none were found in the control group. We observed no founder effect in the Republic of Karelia with respect to the LDL receptor gene mutations. It seems that known mutations from the Finnish population are only a rare cause of the disease in the Karelian FH sample: allele c.925–931del7, known as FH-North Karelia, was found in only one patient, whereas allele FH-Helsinki was not detected at all [11].

### 3.1. Clinical Features and Cholesterol Levels in FH Patients

We analysed the LDL cholesterol levels in patients with definite FH, depending on age (Table 3) and sex (Table 4).

There was a strong overlap between LDL cholesterol levels in different FH age groups. No statistically significant differences between groups were observed in LDL cholesterol levels, despite a tendency for maximal LDL cholesterol values to increase with age in females. Thus, no variation in cut-off points for LDL cholesterol in different FH groups can be recommended.

FH is most often characterized by severe manifestations of atherosclerosis in different locations, especially in the coronary arteries. We observed IHD in 27% of patients with possible FH, in 50% of patients with probable FH, and in 58.6% of patients with definite FH (Table 1). The frequency of IHD in the group with possible FH showed a statistically significant difference compared to the other groups (*p* < 0.01). The same was the true for the frequency of occurrence of myocardial infarction (MI) in the various FH subgroups: 18.5% in possible FH, 26.9% in probable FH, and 30% in definite FH. The difference in the frequency of MI between the groups of probable and possible FH was significant (*p* = 0.0007), as was the difference between the groups of possible and definite FH (*p* = 0.04). The average age of patients with acute MI was significantly lower in the group with definite FH (56.9 ± 2.3 years) than in the groups with probable FH (59.7 ± 3.4 years, *p* < 0.05) and possible FH (68.2 ± 2.0 years, *p* < 0.05).

### 3.2. Mutations in the LDL Receptor Gene and LDL Level

We analysed the relative risk of the presence of mutations in patients with definite FH, depending on the level of LDL cholesterol. The distribution by age and sex and the LDL cholesterol levels of groups of patients with definite FH, depending on the presence or absence of mutations of the LDL receptor, are presented in Table 5.

As can be seen from the data presented in Table 4, no significant differences were identified between the two subgroups in relation to sex, age, or levels of HDL-C and TG. However, significant differences between TC and LDL-C levels were revealed.

**Figure 1:**
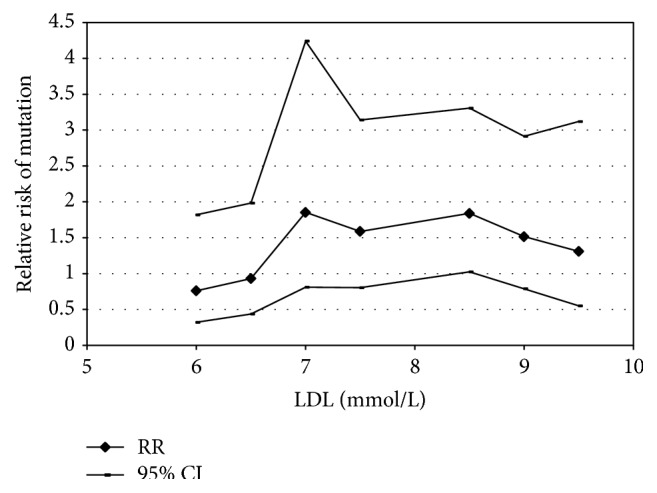
Frequency of mutations in the low density lipoprotein (LDL) receptor in familial hypercholesterolaemia patients according to different LDL cholesterol levels.

As shown in Figure 1, the frequency of mutations in patients with LDL cholesterol levels > 6.5 mmol/L was almost 3 times higher than that in patients with LDL cholesterol levels < 6.5 mmol/L. Using this cut-off point, the odds ratio (OR) was statistically significant: 3.4 (1.4; 8.1), *p* = 0.006.

**Figure 2:**
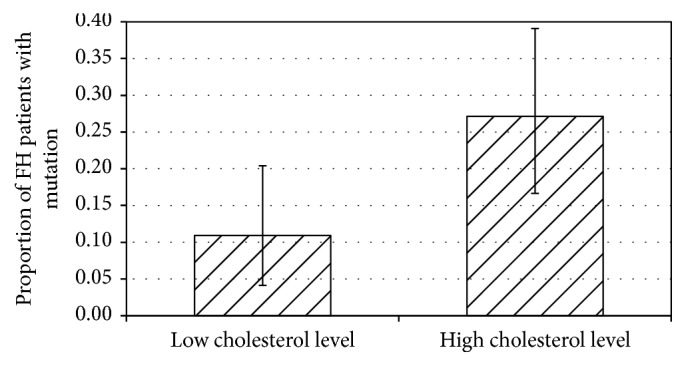
Proportion (with 95% confidence intervals) of familial hypercholesterolaemia patients who had a mutation in relation to low (<6.5 mmol/L) or high (>6.5 mmol/L) low density lipoprotein cholesterol levels. The difference is statistically significant (*p* < 0.03).

Figure 2 demonstrates that only 11% of patients with LDL cholesterol levels < 6.5 mmol/L were carriers of the LDL receptor mutations, whereas this proportion rose to 27% in patients with LDL cholesterol levels > 6.5 mmol/L.

## 4. Discussion

Early diagnosis of FH is very important, because this disease is characterized by the early development of the atherosclerotic process, often at a young age. At the same time, early treatment can stop the progression of the disease and can extend the life expectancy of patients with FH in the general population [5]. The lipid metabolism is subject to genetic control; therefore, diseases such as FH may exhibit ethnic differences [12]. Today, there are several strategies for identifying patients with FH. Most existing scales for the diagnosis of FH are founded on cut-off levels of LDL. These levels are also used in cascade screening [13, 14]. In some cases, this strategy suffers from limitations, because there may be an overlap in the LDL cholesterol cut-off levels between carriers of mutations of the LDL receptor and unaffected relatives. The magnitude of this overlap may vary in different ethnic groups. For example, a significant overlap of these levels has been found in Netherlands, whereas in Japan it is minimal [14, 15]. Nevertheless, using the LDL cut-off levels can work well for identifying patients with FH in some countries (particularly in those countries where there are one or few severe mutations, common to most of the population, such as in Finland or South Africa), although they may be ineffective in other countries that are characterized by genetic diversity [12, 16–18].

An important current question is the role of the genetic method in the diagnosis of FH. Of course, the first step in FH diagnosis is cascade screening and confirmation of the diagnosis of FH with relatives. The level of LDL cholesterol, which is increased in patients with mutations of the LDL receptor, exhibits ethnic differences and differs among different populations. For example, in western Austria a level of LDL cholesterol > 5.9 mmol/L predicts the presence of pathogenic mutations in index patients with maximum sensitivity, whereas for patients with FH from Brazil the corresponding level is 6.5 mmol/L [5]. In our study, the number of FH patients without mutations of the LDL receptor was greatest in the LDL cholesterol range 6–6.5 mmol/L, whereas most patients with the LDL receptor mutation had LDL cholesterol levels of 8.5–9 mmol/L.

In the analysis of sex-specific reference values of LDL in patients with definite FH, we identified the following dynamics: the distribution of values of cholesterol by age and sex showed a characteristic age-related trend towards a gradual increase; however, the substantial overlap did not allow any difference to be established in the cut-off levels of cholesterol for the diagnosis of FH in patients of different ages.

We found significant differences in the TC and LDL cholesterol levels between the group of patients with LDL receptor mutations and those without mutations: TC and LDL cholesterol levels were higher in the group with mutations (Table 5). As regards the inverse relation, we also analysed the rate of detection of mutations of the LDL receptor, depending on the level of LDL cholesterol: in patients with LDL cholesterol levels of more than 6.5 mmol/L the detection rate of mutations of the LDL receptor was 3-fold higher compared to that in patients with LDL cholesterol levels less than 6.5 mmol/L. Thus, genetic testing should first be recommended to FH patients with higher LDL cholesterol values.

## Figures and Tables

**Table 1:** Clinical and biochemical features of patients with heterozygous FH.

	Possible FH (*n* = 126) Group 1	Probable FH (*n* = 26) Group 2	Definite FH (*n* = 102) Group 3	Post hoc analyses
	*M* ± *m*	*M* ± *m*	*M* ± *m*	*p*
Age (years)	50.84 ± 1.57	53.00 ± 3.30	51.56 ± 1.49	>0.05
Male (%)	31.7	42.3	45.9	*p*1-3 < 0.05
BMI (kg/m^2^)	26.93 ± 0.86	28.81 ± 1.79	28.07 ± 0.59	>0.05
Smoking (%)	21.3 ± 4.1	22.2 ± 4.2	24.4 ± 4.3	>0.05
Arterial hypertension (%)	61.4 ± 4.1	65.4 ± 3.8	69.5 ± 4.6	>0.05
Ischemic heart disease (%)	26.8 ± 4.4	50.0 ± 9.6	58.6 ± 4.9	*p*1-3 < 0.05, *p*1-2 < 0.05, *p*2-3 > 0.05
Myocardial infarction (%)	18.5 ± 3.9	26.9 ± 8.5	30.0 ± 4.6	*p*1-3 = 0.007 *p*1-2 = 0.04
DLCN (points)	3.92 ± 0.08	6.08 ± 0.05	11.40 ± 0.48	*p*1-3, *p*2-3, *p*1-2 < 0.05
TC (mmol/L)	8.71 ± 0.09	9.15 ± 0.17	10.14 ± 0.17	*p*1-3 < 0.05, *p*2-3 < 0.05, *p*1-2 = 0.26
LDL-C (mmol/L)	5.81 ± 0.08	6.58 ± 0.17	7.52 ± 0.15	*p*1-2, *p*1-3, *p*2-3 < 0.05
TG (mmol/L)	1.90 ± 0.10	1.96 ± 0.17	1.74 ± 0.08	*p* > 0.05
HDL-C (mmol/L)	1.57 ± 0.05	1.41 ± 0.08	1.49 ± 0.04	*p* > 0.05

LDL-C: low density lipoprotein cholesterol (average rate).

FH: familial hypercholesterolemia.

TC: total cholesterol (average rate).

HDL-C: high density lipoprotein cholesterol (average rate).

TG: triglycerides (average rate).

DLCN: Dutch Lipid Clinic Network.

BMI: body mass index (average rate).

*px*-*y*: *p* value of pairwise difference between Group *x* and Group *y*. The level of significance was *p* < 0.05.

**Table 2:** Newly reported mutations of the low density lipoprotein receptor gene in the Petrozavodsk familial hypercholesterolaemia sample.

Exon/intron	Name of mutation according to genomic DNA LRG_274	Name of mutation according to cDNA	Predicted change in protein amino acid sequence	Rapid method for testing	Number of families (number of patients)
Exon 3	18338 G>C	^*∗*^с.245G>C	*p. (Cys82Ser)* [=p.C61S]	Loss of *HpyCH4*V site	1 (1)

Intron 3	18408 T>G	^*∗*^c.313+2T>G (IVS3+2T>G)	—	New site for *Hpy188I*	1 (1)

Exon 9	28933 G>A	с.1222G>A	*p. (Glu408Lys)* [=p.E387K]	Loss of site for *BssSI*	1 (2)
	29038 T>C	c.1327 T>C	*p. (Trp443Arg)* [=p.W422R]	New site for *MspI*	1 (3)

Exon 13	35725 G>C	^*∗*^с.1859 G>C	*p. (Trp620Ser)* [=p.W599S]	New site for *TaqI*	1 (1)

Note: items in brackets are names of mutations according to traditional nomenclature. This nomenclature does not count amino acids of the LDL receptor signal peptide but starts counting from the first amino acid of the mature receptor. Modern nomenclature starts counting from the first methionine of the signal peptide.

^*∗*^New mutation previously unreported anywhere in the world.

**Table 3:** Low density lipoprotein cholesterol (LDL-C) levels in patients with definite familial hypercholesterolaemia according to age (*n* = 102).

Age	LDL-C (mmol/L)
<20 (*n* = 5)	4.8–6.2
20–30 (*n* = 9)	5.9–8.2
30–40 (*n* = 15)	5.7–9.6
40–50 (*n* = 13)	5.4–9.0
50–60 (*n* = 29)	5.5–11.4
60–70 (*n* = 25)	4.2–11.7
≥70 (*n* = 6)	6.3–12.5

Data are presented as 2.5th–97.5th percentile.

There are no statistically significant differences between groups (*p* = 0.06).

**Table 4:** Low density lipoprotein cholesterol levels (mmol/L) in patients with definite familial hypercholesterolaemia according to age and sex.

Age	Total group	Male (*n* = 47)	Female (*n* = 55)
<30 (*n* = 14)	4.8–8.2	5.9–8.0	4.8–8.2
30–50 (*n* = 28)	5.4–9.6	5.4–9.0	6.3–9.6
50–60 (*n* = 29)	5.5–11.4	5.9–8.7	5.5–11.4
≥60 (*n* = 31)	4.2–12.5	4.2–9.3	6.0–12.5

*Note*. There are no statistically significant differences between groups (*p* = 0.20).

Data are presented as 2.5th–97.5th percentile.

**Table 5:** Comparison of patients with definite familial hypercholesterolaemia with and without LDL receptor gene mutations.

	LDL receptor gene mutations detected (*n* = 22)	LDL receptor gene mutations not detected (*n* = 92)	Statistical significance, *p*
Males, *n* (%)	8 (38.1)	40 (42.1)	0.112
Age, years	46 (28; 55)	53 (36; 63)	0.120
TC, mmol/L	10.3 (8.9; 11.0)	9.1 (8.0; 10.2)	0.013
LDL-C, mmol/L	7.6 (6.4; 8.4)	6,4 (5.4; 7.5)	0.006
HDL-C, mmol/L	1.5 (1.3; 1.7)	1.3 (1.1; 1.6)	0.117
TG, mmol/L	1.6 (1.3; 1.8)	1.7 (1.2; 2.2)	0.300

LDL-C: low density lipoprotein cholesterol.

FH: familial hypercholesterolaemia.

TC: total cholesterol.

HDL-C: high density lipoprotein cholesterol.

TG: triglycerides.

Results are presented as median and interquartile range, unless otherwise specified.
